# Supplementary figures and images for: Polyclonal B cells acquire LCMV antigens in a GP1-dependent manner
Source: PLoS Pathog. 2025 Jul 9;21(7):e1013345. doi: 10.1371/journal.ppat.1013345 (PMC12258595; doi:10.1371/journal.ppat.1013345)

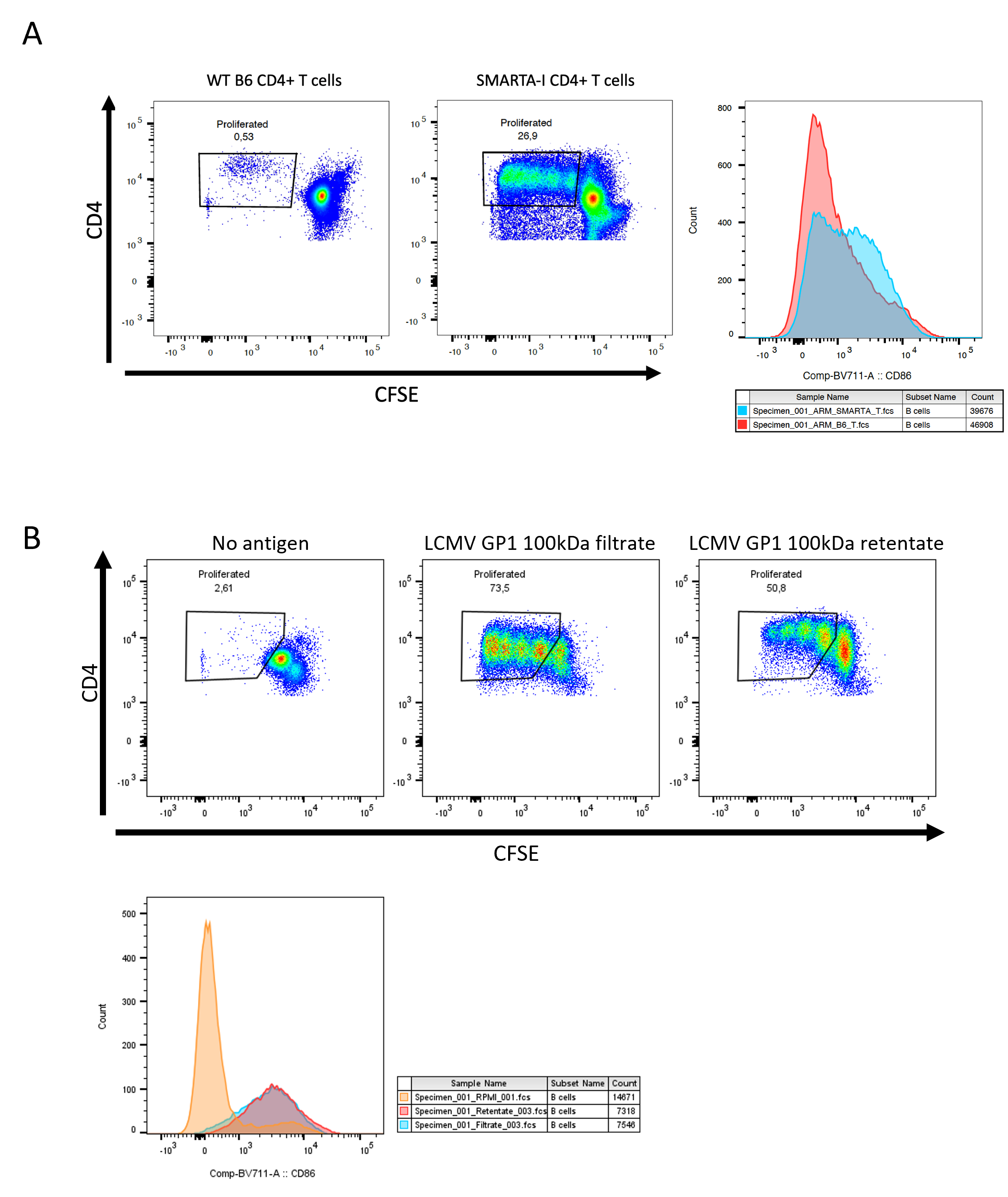

Supplement: S1 Fig — FACS plots of CFSE labeled uninfected wild-type B6 (top left panel) or SMARTA-I (top right panel) CD4+ T cells after 72 hours of co-culture with LCMV-antigen loaded wild-type B cells. Comparative histograms of CD86 expression on B cells exposed to LCMV antigen and co-cultured with CD+ T cells from uninfected wild-type B6 (red) or SMARTA-I (blue) mice (bottom panel). (B) Filter validation experiments. FACS plots showing CFSE labelled SMARTA-I CD4+ T cells after 72 hours of co-culture with B cells loaded with either RPMI (top left panel), LCMV WE GP1 100kDa filtrate (top middle panel), or LCMV WE GP1 100kDa retentate (top right panel). Comparative histograms of CD86 expression on B cells exposed to RPMI (orange), LCMV WE GP1 filtrate (blue), or retentate (red), and co-cultured with CD4+ T cells. (TIF) [file ppat.1013345.s001.tif]

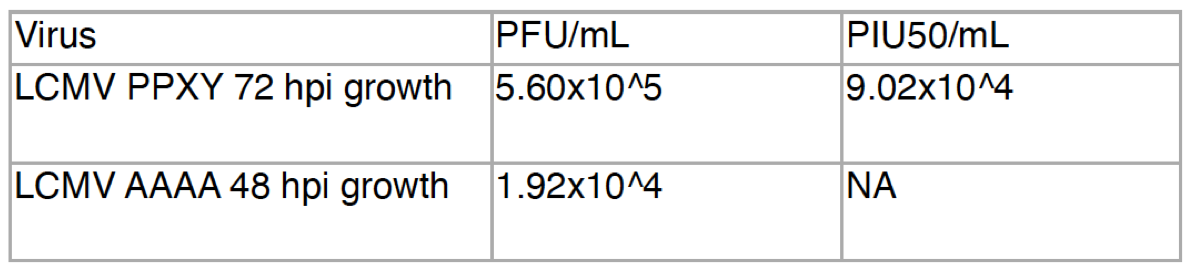

Supplement: S2 Fig — Viral titration (left) to quantify viral concentration in plaque forming units (PFU) and interference assay (right) to quantify DIP concentration in plaque interfering units50 (PIU50/ml) in wild-type LCMV (PPXY) (top) vs mutated LCMV (AAAA) (bottom) samples. (TIF) [file ppat.1013345.s002.tif]

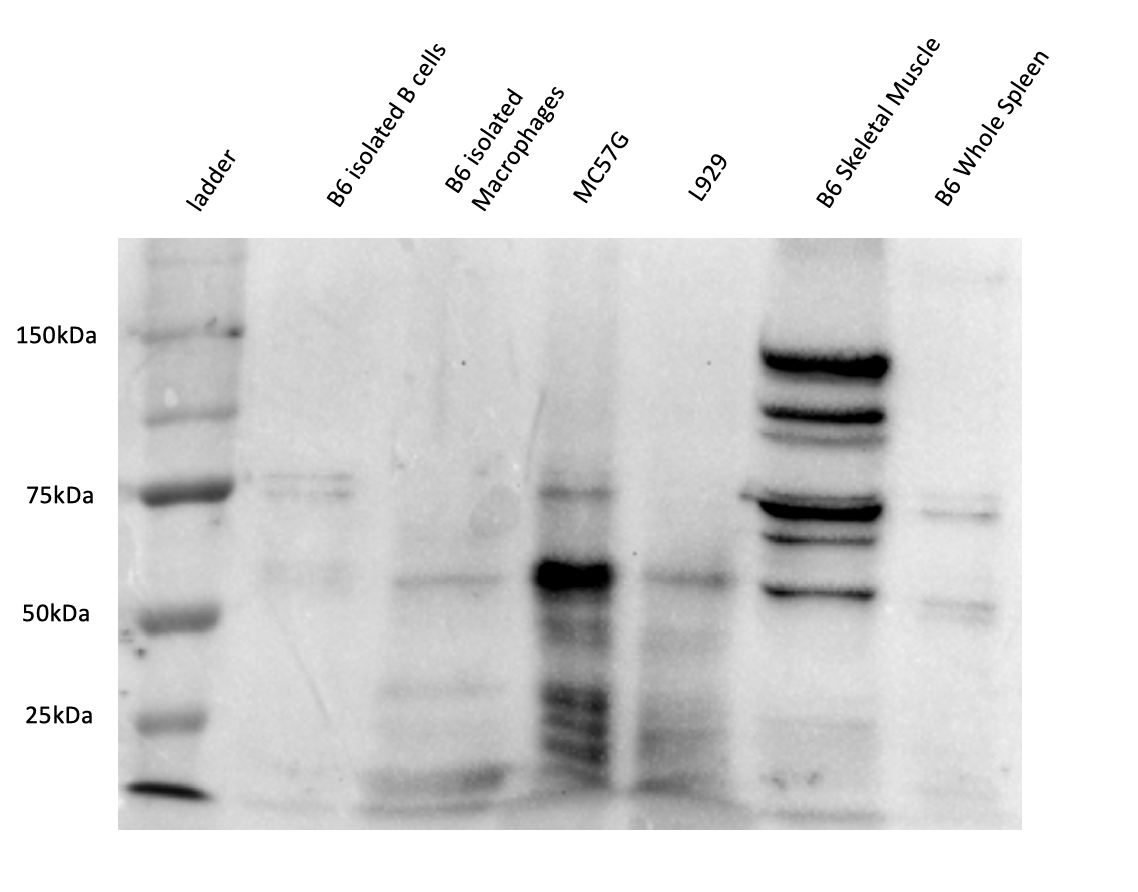

Supplement: S3 Fig — A western blot using the VIA4–1 antibody which recognizes glycosylated α-dystroglycan was performed on lysates from various tissues isolated from C57BL/6 (B6) mice or from murine cell lines commonly used to grow LCMV. The size of α-DG increases due to extensive O-glycosylation and variable N-glycosylation in different tissues. (TIF) [file ppat.1013345.s003.tif]

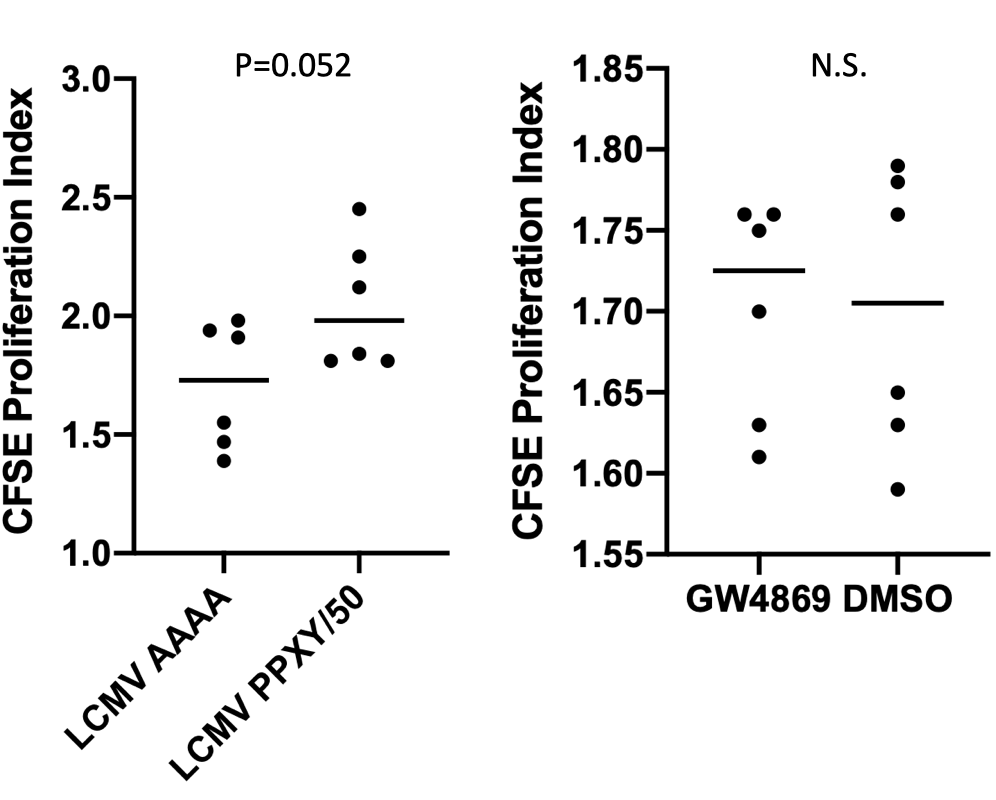

Supplement: S5 Fig — CFSE loss for CD4+ SMARTA-I T cells was measured in FACS following co-culture with antigen exposed B cells. Proliferation Index analysis was performed using the Proliferation tool in FlowJo. (TIF) [file ppat.1013345.s005.tif]
