# Supplementary material for: Polyclonal B cells acquire LCMV antigens in a GP1-dependent manner
Source: PLoS Pathog. 2025 Jul 9;21(7):e1013345. doi: 10.1371/journal.ppat.1013345 (PMC12258595; doi:10.1371/journal.ppat.1013345)
Supplement: Attachment — Submitted filename: Response to Reviewers.docx [file ppat.1013345.s007.docx]

**Reviewers' Comments:**

**Part I - Summary**

Reviewer #1: They report that B cells are accessing LCMV viral antigen at larger than 200kda sizes in a receptor mediated manner. They show that the B cells can present the antigen even if the virus is not infectious, and this is not dependent on IDs or exosomes. The novel finding was that B cells are acquiring antigen from articles which are larger than 200kda. This opens the door to many more questions about this process but that could be for another project.

Reviewer #2: In this study, Chamberlain and colleague attempt to gain a better understanding of how B cells acquire antigens from LCMV in order to induce hypergammaglobulinemia. They use a combination of techniques and come to the conclusion that receptor mediated endocytosis is a major pathway explaining how B cells acquire antigen from LCMV. The experiments are well-controlled and generally support the conclusions. There are a few concerns the dampen the enthusiasm from the study in its current form. Most are minor concerns but the main concern is that while they are trying to link these studies back to HGG this is never tested in the current manuscript. Without establishing this link, it is this reviewer's opinion that the study would be better suited for a more specialized journal.

Reviewer #3: Chronic viral infections induce hyperglobulinemia in a T cell-dependent but B cell antigen receptor-independent manner, suggesting that viral antigens may be acquired via alternate means by polyclonal B cells. in In this study, the authors investigate novel mechanisms of antigen acquisition by polyclonal B cells using LCMV as a model system. They use T cell activation as the primary readout of antigens acquired and presented by B cells, and show that LCMV antigens are accessible to B cells in vitro from supernatants of infected cells (likely through fluid uptake, i.e., pinocytosis), and thus direct productive infection of B cells is not required. Further, the authors show that the size of antigens that are taken up by B cells is >200 kDa (likely membrane-bound vesicles but not free GP-1 monomers or trimers) as the retentates of culture supernatants passed through 100 and 200 kDa cut-off filters induced T cell activation and cytokine production but <100 and <200 kDa filtrates did not. They go on to demonstrate that antigens acquired by polyclonal B cells in this manner are capable of inducing T cell activation in vivo when such B cells are transferred into LCMV-infected mice. Finally, the authors suggest that binding of LCMV GP-1 to a potential receptor on B cells enables antigen uptake as the presence of an LCMV neutralizing antibody abrogates activation of T cells. The experiments are conducted logically and methodically. The findings open new avenues for exploring antigen uptake by polyclonal B cells in other human viral infections, and potentially identifying ways to block BCR-independent modes of uptake.

We sincerely thank all three reviewers for their careful and thorough review of our manuscript and positive comments on the study. We have addressed all of their questions and believe that this has considerably strengthened the conclusions of the study.

**Part I -** The following weaknesses must be addressed to strengthen the conclusions:

1. The authors use mechanical size separation using 100 kDa and 200 kDa cut-off filters to identify the size of antigens taken up by B cells. In both cases, retentates of the filtration system contained the antigen that is taken up and presented to T cells. Depending on the material of the filters used, is it possible that the antigens (especially free proteins) are simply adsorbed on the filters and thus not available at high enough concentration in the filtrates? Could these antigens be detected on the top surface of the filters to ensure that this is not the case?

This is an important point, as it is crucial to validate that our findings truly reflect the nature of the antigen rather than overlooked technical limitations. To address this, we assessed the ability of recombinant LCMV WE GP to pass through the filters used in our study. We performed filtration under the same conditions as in our previous experiments and used both the filtrate and retentate in subsequent antigen presentation assays. The results have been included in the revised manuscript (Fig. S1B) and are discussed in the Results section (**page 9-10, lines 200–207**).

2. In most experiments, the authors assay antigen uptake by measuring T cell proliferation, CD86 upregulation on B cells, and cytokine levels in the B-T co-cultures. What is the mechanism of CD86 upregulation in a scenario where the BCR is not involved? Are there other changes in B cell properties upon BCR-independent LCMV antigen acquisition – signaling, transcriptional activation, in vitro proliferation?

We thank the reviewer for this comment, as this is an important question. In our assay B cells upregulate CD86 in response to stimulation from T cells via MHC-II/TCR engagement as B cells without antigen do not experience increased CD86 expression despite contact with T cells. Likewise, B cells given LCMV antigen do not upregulate CD86 when co-cultured with polyclonal CD4 T cells from uninfected WT mice instead of SMARTA-I mice, we added and discussed data showing this to Fig S1 of the revised manuscript (**see pages 17-18, lines 387-394**). IFNAR signalling is another example of a BCR-independent mechanism of CD86 upregulation which we frequently detect in total splenic B cells from LCMV infected mice, for this reason we do not include B cell CD86 in Fig 5 data as all B cells in LCMV infected mice are CD86 high in response to elevated IFN-I levels (absent in our *in vitro* assay). Thus, we did not see any changes in the properties of B cells we measured as a direct consequence of exposure to LCMV antigen, rather only upon antigen presentation to cognate T cells. Since cognate T cell help is well established as a requisite for HGG in LCMV our study focuses on antigen presentation and B cell properties most closely associated with CD4+ T cell interaction. We have elaborated on our reasoning in the discussion of the revised manuscript (**see pages 18-19, lines 401-412**).

Regarding changes to B cell properties upon co-culture with T cells, we measured B cell proliferation *in vivo* after adoptive transfer to infected mice in Fig. 5, otherwise we also measured B cell upregulation of MHC-II which we have added to the supplementary figures in Fig S4 of the revised manuscript (**see page 13, lines 276-277**). It should be noted however that MHC-II expression was often variable in our control group (B cells given uninfected cell supernatant) and therefore despite sometimes interesting results like those in Fig S4 we found MHC-II less reliable as a readout compared to our other metrics. Similarly, in our very early experiments we also included CD69 data on B cells but as this is a very early activation marker and we look at B cells three days after co-culture, although CD69 upregulation was detectable we found it to be much more variable than CD86 and therefore to be less reliable in our experimental setting.

Our objective was to create a minimalist assay to determine under what conditions B cells could acquire antigens. We chose to focus on a few of most well cited metrics for B cell activation: CD86 and IL-6 production. While these are far from exhaustive we found them to be robust and meet the needs of our assays.

3. In Figure 5, the authors show that polyclonal B cells proliferate after presenting antigens acquired in vitro to T cells in LCMV-infected mice. It is not clear what signal these B cells are responding to.

In Figure 5 we show that B cells adoptively transferred into LCMV infected mice that did not receive LCMV antigen *in vitro* prior to their transfer do not proliferate while B cells that did receive LCMV antigen *in vitro* prior to their transfer did proliferate. In Figure S1 we show that B cells given LCMV antigen alone have no effect if the CD4 T cells in their co-culture are not specific to LCMV. Taken together as B cell proliferation in Figure 5 is antigen dependent we interpret our results to mean that B cells are responding to signals acquired from T cell help *in vivo* in a similar manner as they respond to the same help from SMARTA-I T cells *in vitro*. We have added a more detailed discussion on figure 5 to our revised manuscript to clarify this point (**see page 18, lines 401-412**).

4. As anti-LCMV antibodies block Ag presentation by polyclonal B cells in vitro, it is suggested that a GP-1 receptor may exist on B cells that mediates uptake of viral antigens (membrane vesicles?) via endocytosis. Are GP-1 or viral particles or membrane vesicles bearing viral antigens detectable inside the B cell endosomes? There are ways to block endocytosis and should be used to test if this is the only mechanism or one of the mechanisms by which polyclonal B cells acquire antigen in the in vitro system utilized in this study. This is important as pinocytosis is also considered as a potential mechanism of antigen uptake.

While we did make attempts to block endocytosis in B cells using chlorpromazine to block clathrin-mediated endocytosis we found that primary B cells did not survive inhibition for more than a few hours making it difficult to use in our multi-day assay. We also could not find a way to selectively inhibit pinocytosis without also affecting receptor mediated endocytosis and so these assays were abandoned and our unsuccessful attempts were not included in our manuscript.

**Part II – Major Issues: Key Experiments Required for Acceptance**

Reviewer #2: The authors never show that blocking receptor mediated endocytosis lead to any impact on HGG which the major rationale for these studies. Without this link, the enthusiasm for this study is significantly dampened

We thank the reviewer for this important comment. Blocking receptor mediated endocytosis is an approach that was considered early on by the authors though, frustratingly, proved to have significant limitations. Pinocytosis and receptor mediated endocytosis use various, and often overlapping, mechanisms of entry (ex: clathrin-mediated endocytosis [CME]) therefore, to our knowledge, it is not possible to specifically block the various mechanisms of receptor mediated endocytosis without also blocking pinocytosis, thereby rendering such an assay inconclusive with regards to our hypothesis. Nonetheless, we still initially made attempts to block CME with chlorpromazine in our *in vitro* model though, unfortunately, we found that B cells did not respond well to chlorpromazine *in vitro* for the timespans required by our assays. CME blocking through chlorpromazine has been shown to reduce viral entry to permissive cells in HCV, SARS-CoV2 and MHV-2 Stip 2020 (doi: 10.1177/0706743720934997). Similarly, systemic blockade of receptor-mediated endocytosis of LCMV risks interfering with infection of permissive cells *in vivo*, thereby making it impossible to distinguish effects on B cell acquisition from those caused by reduced virulence. Along the same reasoning, the KL25 neutralizing antibody we use *in vitro* is known to reduce viral titers *in vivo* and facilitate viral clearance in mice (doi: 10.1016/j.celrep.2022.110303). It is not possible to differentiate the consequences of reduced viral titers and reduced B cell access to antigen as they pertain to HGG *in vivo* using KL25 as we have *in vitro*. Since blocking endocytosis is an avenue that was considered by both the authors and the reviewer, we have added the above points to the discussion of our revised manuscript so that the reader will also benefit from understanding the limitations of this approach (**see page 23, lines 516-523**).

Despite the aforementioned challenges in inhibiting endocytosis, we are grateful for this question, as it highlighted that we had not sufficiently emphasized important findings on which our project is based. We chose to focus on the antigen acquisition aspect of HGG as others have already shown that once polyclonal B cells have acquired LCMV antigen in a BCR-independent manner they will proliferate and produce polyclonal antibodies, see Jellison *et al* 2007 (DOI: 10.1002/eji.200636516). Their study shows how the LCMV GP61 MHC-II epitope directly loaded onto B cells drives polyclonal antibody production when transferred *in vivo*. Our manuscript ends where Jellison *et al* begins and complements their findings by showing how LCMV antigen is acquired in the first place. We have added emphasis on this point in the Introduction and Discussion of our resubmitted manuscript (**see page 5, lines 93-96 and page 24, lines 527-530, respectively**).

Reviewer #3: The following concerns must be addressed through experimentation and discussion:

1. The authors use mechanical size separation using 100 kDa and 200 kDa cut-off filters to identify the size of antigens taken up by B cells. In both cases, retentates of the filtration system contained the antigen that is taken up and presented to T cells. Depending on the material of the filters used, is it possible that the antigens (especially free proteins) are simply adsorbed on the filters and thus not available at high enough concentration in the filtrates? Could these antigens be detected on the top surface of the filters to ensure that this is not the case?

Answered above, see part 1, comment #1.

2. In most experiments, the authors assay antigen uptake by measuring T cell proliferation, CD86 upregulation on B cells, and cytokine levels in the B-T co-cultures. What is the mechanism of CD86 upregulation in a scenario where the BCR is not involved? Are there other changes in B cell properties upon BCR-independent LCMV antigen acquisition – signaling, transcriptional activation, in vitro proliferation?

Answered above, see part 1, comment #2.

3. As anti-LCMV antibodies block Ag presentation by polyclonal B cells in vitro, it is suggested that a GP-1 receptor may exist on B cells that mediates uptake of viral antigens (membrane vesicles?) via endocytosis. Are GP-1 or viral particles or membrane vesicles bearing viral antigens detectable inside the B cell endosomes? There are ways to block endocytosis and should be used to test if this is the only mechanism or one of the mechanisms by which polyclonal B cells acquire antigen in the in vitro system utilized in this study. This is important as pinocytosis is also considered as a potential mechanism of antigen uptake.

Answered above, see part 1, comment #4.

**Part III – Minor Issues: Editorial and Data Presentation Modifications**
Please use this section for editorial suggestions as well as relatively minor modifications of existing data that would enhance clarity.

Reviewer #1: Figure 1. Should the statistical analysis be unpaired T test when comparing more than 2 variables? You may get the same answer, but the ANOVA and posttest would be a sounder statistical analysis.

Our statistical analysis was carried out to independently compare each condition to Mock (the negative control/null hypothesis) and therefore were always between 2 variables. While statistics could be applied among the other variables (ex. between unfiltered and 3x retentate conditions in Fig 1.) these were not comparisons that were relevant for the purpose of our assays and therefore were neither calculated nor discussed.

Figure 6. Because the sups from WT LCMV infected cells had a 50-fold higher titer than the LCMV-AAAA, you diluted it to control for infectious content but does your dilution now compare a very low DI level to an undetectable DI level? Thus, giving the results observed?

Since we could not rule out whole infectious virions as a potential source of viral antigen in our assay, it was necessary to ensure viral titers between groups were as close as possible in order to make inferences on comparisons between these groups. DI levels in WT LCMV are 9.02x10^4 PIU50/mL (Fig. S2) prior to dilution and so we expect roughly 1804 PIU50/mL at a 1/50 dilution. However, regardless of the dilution of the WT LCMV, we should not expect to see any metrics of proliferation in LCMV-AAAA if DI were indeed minimally required for B cell antigen acquisition, which is not the case. It should also be noted that while not statistically significant, all antigen presentation metrics except IL-6 were slightly higher in the AAAA DI null mutant compared to diluted WT PPXY LCMV further supporting our conclusion that DI is not minimally required/redundant to other available forms of antigen.

Figure 7. Is there a reason for switching to the Cl-13 strain in this experiment?

We are grateful for this question as omitting our justification was admittedly an oversight. The GP1 of the ARM strain used in our other experiments is not neutralized or bound by KL25 mAb. KL25 neutralizes the WE strain of LCMV however in North America the WE strain of LCMV is classified as a biosafety level (BSL) 3 pathogen instead of level BSL 2 pathogen like LCMV strains ARM and Cl-13. We used the recombinant LCMV rCl-13WE-GP because it can be bound by KL25 and is BSL 2 rendering it safe to use in our level 2 research facility. We produced rCl-13WE-GP in the same cell line we used to produce ARM LCMV. The Cl-13 variant of ARM differs from the parental ARM (53b) in two mutations, the first in the polymerase and the second in the glycoprotein. Since rCl-13WE-GP expresses the WE glycoprotein is does not harbour the Cl-13 GP mutation. Finally, as we determined that intracellular infection was not minimally required for antigen presentation we did not expect the polymerase mutation to have an effect in our *in-vitro* assays. Consistent with our reasoning, we did not observe any biological differences between ARM and rCl-13WE-GP in the context of our *in-vitro* assays other than the ability to bind KL25. We have included our justification for changing LCMV strains in the revised manuscript (**see page 16-17, lines 360-366**)**.**

Reviewer #2: In figure 2 it appears that the majority of cells have divided at least once but this is ignored and not discussed. This is either due to some baseline proliferation or poor CFSE staining but this needs to be addressed.

This is a good observation and an oversight on our part, we have added a sentence discussing this in the results section of our revised manuscript (**see page 9, lines 187-190**). In brief, we believe this observation in Figure 2 is due to “proliferation-independent CFSE loss” similar to what is described in Figure 2 of Tario *et al* 2011 (DOI: 10.1007/978-1-61737-950-5_7).

The data on proliferation is only analyzed based on the percentage of cells that have divided but this is problematic. Measuring a proliferation index that takes into account how many times the cells have divided would be much more informative and would likely detect some of the more minor differences. For example, in figure 6A it appears that while a similar percentages of cells have initiated division, more cells have undergone extensive division in the PPXY group. Since some of the cells that have completely diluted CFSE may have undergone several more rounds of division, these data are important. This analysis should be provided throughout the study.

We initially decided to use percentage of cell divided as in our first set of antigen presentation assays (Fig 2) we found that conditions either could induce proliferation or could not without any middle ground. in Fig 7 we show that KL25 significantly impacts the percentage of proliferating demonstrating that our method of measuring proliferation can effectively capture instances of significant inhibition. While we do not expect any proliferation to occur in Figure 6B if DI are indeed minimally required, we agree with the reviewer that both figures 6A and 6B would benefit from an analysis of their proliferation index in addition to proliferation percentage and have included these results in Fig S5 of our reviewed manuscript. Our analysis confirms the reviewer’s observation of a difference in proliferation index in Fig. 6A however, though very close (P=0.052), does not attain statistical significance. Because the difference is so near to statistical significance we though it still merits discussion and have added to the revised manuscript that we believe this result supports the idea that DI while not minimally required are still redundant sources of antigen for B cells (**see page 15, lines 325-329**).

In general, we are careful in our discussion to make clear that we do not interpret our results to mean that exosomes and DIs are not potential sources of antigen rather that they are not the exclusive form of antigen available to B cells, thus, importantly demonstrating that the virus itself is a possible source of antigen. We do suppose that exosomes and DI, being morphologically very much similar particles to whole infectious virion, are sources of antigens for B cells and can act as redundant sources of antigen with whole virus. Furthermore, in our UV killed LCMV sample there would be no biological difference between whole virion and DI to our knowledge. For the purpose of our study however the results in Figure 6 are important to show that whole virus cannot be excluded as a source of antigen.

Reviewer #3: (No Response)
